# Crystal structure of 5-benzoyl-2,4-di­phenyl-4,5-di­hydro­furan-3-carbo­nitrile

**DOI:** 10.1107/S2056989015014887

**Published:** 2015-08-15

**Authors:** V. Rajni Swamy, R.V. Krishnakumar, S. Sivakumar, N. Srinivasan, R. Ranjith Kumar

**Affiliations:** a Department of Physics, Thiagarajar College, Madurai 625 009, Tamil Nadu, India; b School of Chemistry, Madurai Kamaraj University, Madurai 625 021, Tamil Nadu, India

**Keywords:** crystal structure, furan, carbo­nitrile, hydrogen bond

## Abstract

In the title compound, C_24_H_17_NO_2_, the carbonyl O atom of the benzoyl group is *cis* with respect to the furanyl O atom, and the associated O—C—C—O torsion angle is 4.62 (19)°. The puckering of the dihydro­furan ring is close to twisted (^4^ *T*_5_), with parameters *Q* = 0.1856 (16) Å and φ = 313.5 (5)°. Mol­ecules are inter­connected *via* a C—H⋯N and a C—H⋯O hydrogen bond, leading to layers parallel to the (200) plane and characterized by *R*_4_^4^(28) and *R*_4_^4^(36) graph-set motifs. The furan O atom does not participate in inter­molecular hydrogen bonding. The crystal lattice encompasses a solvent-accessible void of 24.7 (8) Å^3^.

## Related literature   

For biological activity of di­hydro­furans, see: Simmonds *et al.* (1990 ▸); Gebbinck *et al.* (1999 ▸); Ley *et al.* (1987 ▸); Kumar *et al.* (2003 ▸); Pour *et al.* (2003 ▸); Loğoğlu *et al.* (2010 ▸). For Cambridge Structural Database, see: Groom & Allen (2014 ▸). For graph-set motifs, see: Bernstein *et al.* (1995 ▸). For puckering of rings, see: Cremer & Pople (1975 ▸). For related structures, see: Rajni Swamy *et al.* (2012 ▸); Suresh *et al.* (2012*a* ▸,*b* ▸,*c* ▸).
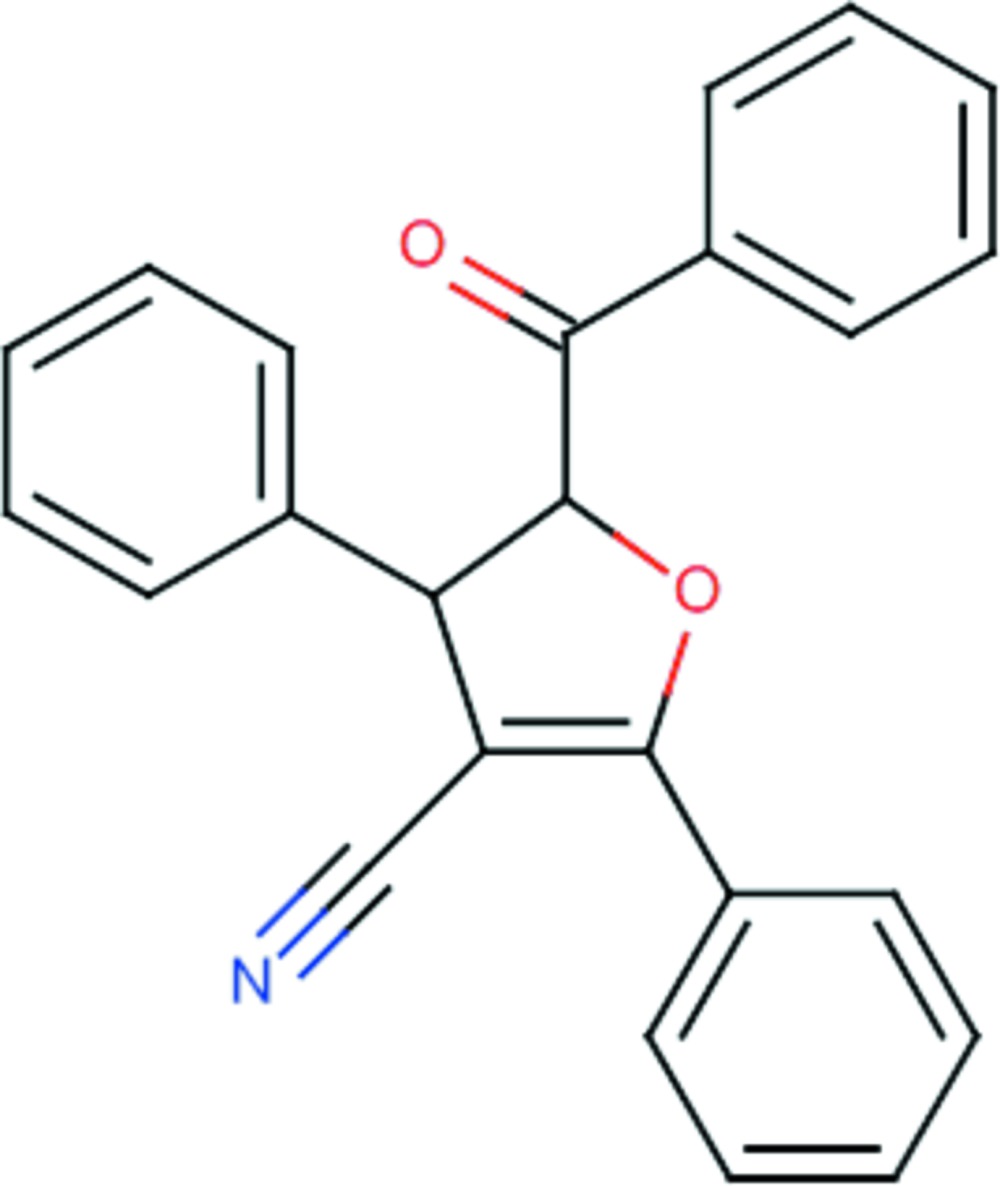


## Experimental   

### Crystal data  

C_24_H_17_NO_2_
*M* *_r_* = 351.38
Monoclinic, 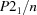

*a* = 10.0704 (7) Å
*b* = 15.7994 (12) Å
*c* = 11.8632 (9) Å
β = 98.886 (3)°
*V* = 1864.9 (2) Å^3^
*Z* = 4
Mo *K*α radiation
μ = 0.08 mm^−1^
*T* = 298 K
0.35 × 0.24 × 0.08 mm

### Data collection  

Bruker SMART APEXII CCD diffractometer
Absorption correction: multi-scan (*SADABS*; Bruker, 2009 ▸) *T*_min_ = 0.973, *T*_max_ = 0.994
34345 measured reflections
4648 independent reflections
2305 reflections with *I* > 2σ(*I*)
*R*_int_ = 0.063

### Refinement  

*R*[*F* ^2^ > 2σ(*F* ^2^)] = 0.050
*wR*(*F* ^2^) = 0.147
*S* = 0.99
4648 reflections
245 parameters
H-atom parameters constrained
Δρ_max_ = 0.15 e Å^−3^
Δρ_min_ = −0.16 e Å^−3^

### 

Data collection: *APEX2* (Bruker, 2009 ▸); cell refinement: *SAINT* (Bruker, 2009 ▸); data reduction: *SAINT*; program(s) used to solve structure: *SHELXS2013* (Sheldrick, 2008 ▸); program(s) used to refine structure: *SHELXL2014* (Sheldrick, 2015 ▸); molecular graphics: *PLATON* (Spek, 2009 ▸); software used to prepare material for publication: *publCIF* (Westrip, 2010 ▸).

## Supplementary Material

Crystal structure: contains datablock(s) I, New_Global_Publ_Block. DOI: 10.1107/S2056989015014887/xu5865sup1.cif

Structure factors: contains datablock(s) I. DOI: 10.1107/S2056989015014887/xu5865Isup2.hkl

Supporting information file. DOI: 10.1107/S2056989015014887/xu5865Isup3.cml

**Fig. 1.**
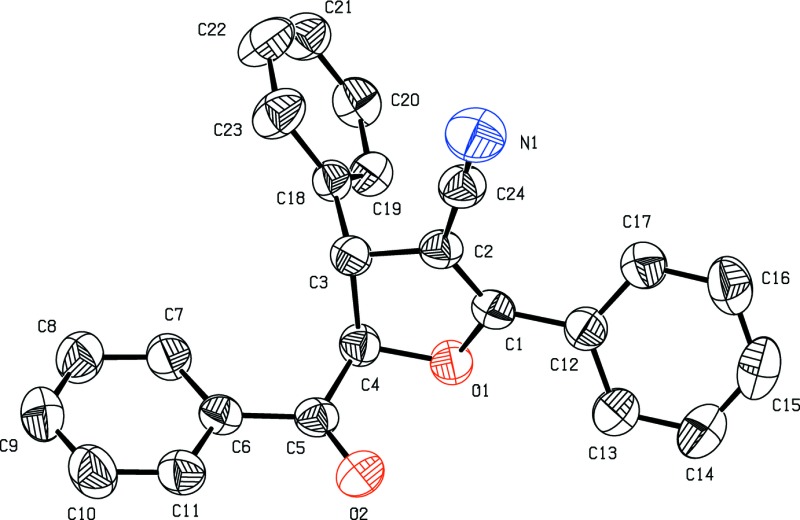
The molecular structure of (I), showing the atom-numbering scheme and displacement ellipsoids drawn at the 50% probability level. H atoms have been omitted for clarity.

**Fig. 2.**
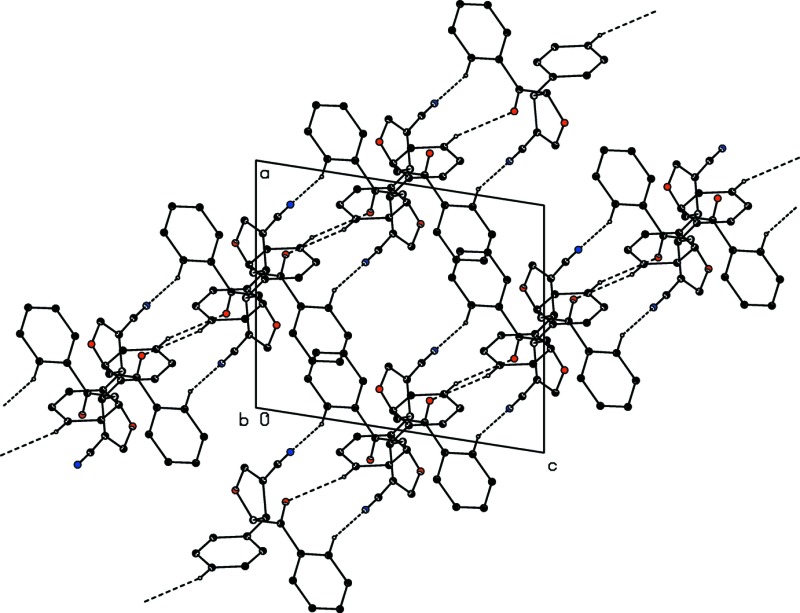
A view down the b axis, showing molecules interconnected via C—H···N and a C—H···O hydrogen bonds, leading to layers parallel to the (200) plane.

. DOI: 10.1107/S2056989015014887/xu5865fig1.tif
Mol­ecular structure of (I) showing the atom numbering scheme and displacement ellipsoids drawn at the 50% probability level. Hydrogen atoms and atoms of minor disordered components omitted for clarity.

b via . DOI: 10.1107/S2056989015014887/xu5865fig2.tif
A view down the *b* axis showing mol­ecules inter­connected *via* a C—H⋯N and a C—H⋯O hydrogen-bond leading to layers parallel to the (200) plane.

**Fig. 3.**
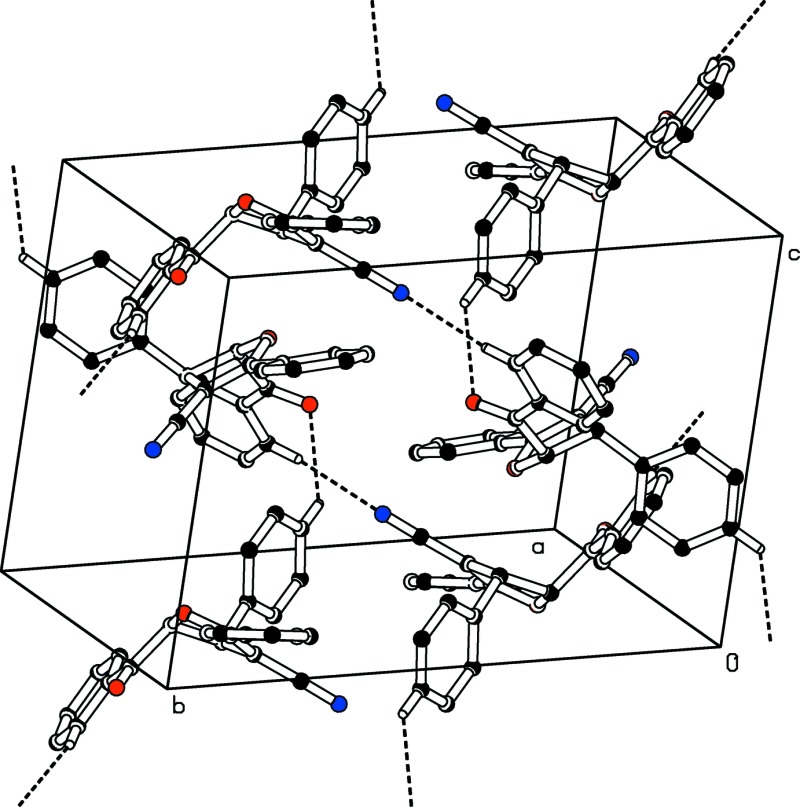
A view of the molecules of the unit cell, showing C—H···N and C—H···O hydrogen bonds.

. DOI: 10.1107/S2056989015014887/xu5865fig3.tif
A view of the mol­ecules of the unit cell showing C—H⋯N and C—H⋯O hydrogen bonds.

CCDC reference: 1023392

Additional supporting information:  crystallographic information; 3D view; checkCIF report

## Figures and Tables

**Table 1:** Hydrogen-bond geometry (, )

*D*H*A*	*D*H	H*A*	*D* *A*	*D*H*A*
C11H11N1^i^	0.93	2.63	3.487(2)	154
C21H21O2^ii^	0.93	2.58	3.277(2)	132

Symmetry codes: (i) 
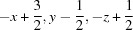
; (ii) 
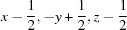
.

## supplementary crystallographic information

### S1. Introduction

The title compound 5-benzoyl-2,4-di­phenyl-4,5-di­hydro­furan-3-carbo­nitrile (I) is a di­hydro­furan carbo­nitrile derivative. Di­hydro­furans belong to an important class of heterocycles and are known for distinct insect anti­feedant activities (Gebbinck *et al.*, 1999). Di­hydro­furans have also been found to possess anti­fungal (Pour *et al.*, 2003) and anti-inflammatory properties (Kumar *et al.*, 2003). The di­hydro­furan derivatives with their reactive functional groups like meth­oxy, carbonyl etc. may prove to be promising candidates for the synthesis of novel heterocyclic compounds. The *in vitro* anti­bacterial and anti­fungal activities of some furan derivatives, specifically, 4,5-di­hydro­furan-3-carbo­nitriles were investigated against some bacteria and fungi and were found to show activity against bacteria better than some known anti­biotics (Loğoğlu *et al.*, 2010).

### S2.1. Synthesis and crystallization

A mixture of benzoyl­aceto­nitrile (1.0 mmol), benzaldehyde (1.0 mmol) and imidazolium salt (1-methyl-3-(2-oxo-2-phenyl­ethyl)-1H-imidazol-3-ium bromide) (1.0 mmol) were dissolved in EtOH (10 mL) then Et_3_N(tri­ethyl­amine) (1.0 mmol) was added slowly and refluxed on a water bath. The consumption of starting material was monitored by TLC. After 3hours the reaction mixture was cooled and the precipitated solid product was filtered and washed. Crystals of (1) suitable for diffraction were obtained.

### S2.2. Refinement

All H atoms were included into the model at geometrically calculated positions (C—H target distance = 0.98 Å for methine H atoms and 0.93 Å for all other aromatic H atoms) and refined using a riding model. The *U*_iso_ values of all H atoms were constrained to 1.2 times *U*_eq_ of the respective atom to which the H atom binds.

### S3. Results and discussion

The carbonyl O of the benzoyl group is cis with the furanyl O atom with the value of the associated torsion angle being 4.62°. The puckering of the hydro­furan ring is close to twisted (^4^*T*_5_) with parameters *Q* = 0.1856 (16) Å and φ = 313.5 (5)°. The angle between the mean plane about the furan ring atoms and the 2-phenyl ring is 22.5 (1)° while those with respect to the 4-phenyl and the 5-benzoyl group atoms are 88.6 (1) and 78.4 (1)°, respectively.

A search in the CSD [version 5.53, update 3, May 2013] for organic nonpolymeric single crystal structures for which 3D coordinates determined with no disorder, no ions and no other errors with R-factors less than 0.05 revealed 6287 structures of which 2498 had the furan O involved in C—H···O inter­actions with the H···A ranging from 2.127 to 2.720 Å. Also, four structures bearing close relationships to the title compound with refcodes LEFJUD (Suresh *et al.*, 2012a), YAXGOV (Suresh *et al.*, 2012b), ZARBEB (Suresh *et al.*, 2012c) and EDUZAG (Rajni Rajni *et al.*, 2012) were found. These structures differ by an indole ring replaced by a phenyl in the title compound and inter­estingly none display any change in the crystal system or lattice type. However, there are drastic differences in the inter­molecular inter­action patterns. The molecules are inter­connected via a C—H···N and a C—H···O hydrogen-bond leading to a layers parallel to the (200) plane and characterized by R_4_^4^(28) and R_4_^4^(36) graph-set motifs. The furan O atom does not participate in the inter­molecular hydrogen bonding. The crystal lattice encompasses a solvent accessible void of 24.7 (8) Å^3^.

### Crystal data

**Table:**

C_24_H_17_NO_2_	*F*(000) = 736
*M**_r_* = 351.38	*D*_x_ = 1.252 Mg m^−^^3^
Monoclinic, *P*2_1_/*n*	Mo *K*α radiation, λ = 0.71073 Å
*a* = 10.0704 (7) Å	Cell parameters from 2305 reflections
*b* = 15.7994 (12) Å	θ = 2.2–28.6°
*c* = 11.8632 (9) Å	µ = 0.08 mm^−^^1^
β = 98.886 (3)°	*T* = 298 K
*V* = 1864.9 (2) Å^3^	Block, colourless
*Z* = 4	0.35 × 0.24 × 0.08 mm

### Data collection

**Table:**

Bruker SMART APEXII CCD diffractometer	2305 reflections with *I* > 2σ(*I*)
Radiation source: fine-focus sealed tube	*R*_int_ = 0.063
ω and φ scans	θ_max_ = 28.6°, θ_min_ = 2.2°
Absorption correction: multi-scan (*SADABS*; Bruker, 2009)	*h* = −13→12
*T*_min_ = 0.973, *T*_max_ = 0.994	*k* = −21→21
34345 measured reflections	*l* = −15→15
4648 independent reflections	

### Refinement

**Table:**

Refinement on *F*^2^	Hydrogen site location: inferred from neighbouring sites
Least-squares matrix: full	H-atom parameters constrained
*R*[*F*^2^ > 2σ(*F*^2^)] = 0.050	*w* = 1/[σ^2^(*F*_o_^2^) + (0.0717*P*)^2^] where *P* = (*F*_o_^2^ + 2*F*_c_^2^)/3
*wR*(*F*^2^) = 0.147	(Δ/σ)_max_ < 0.001
*S* = 0.99	Δρ_max_ = 0.15 e Å^−^^3^
4648 reflections	Δρ_min_ = −0.16 e Å^−^^3^
245 parameters	Extinction correction: *SHELXL2014* (Sheldrick 2015), Fc^*^=kFc[1+0.001xFc^2^λ^3^/sin(2θ)]^-1/4^
0 restraints	Extinction coefficient: 0.0097 (17)

### Special details

**Table:**

Geometry. All e.s.d.'s (except the e.s.d. in the dihedral angle between two l.s. planes) are estimated using the full covariance matrix. The cell e.s.d.'s are taken into account individually in the estimation of e.s.d.'s in distances, angles and torsion angles; correlations between e.s.d.'s in cell parameters are only used when they are defined by crystal symmetry. An approximate (isotropic) treatment of cell e.s.d.'s is used for estimating e.s.d.'s involving l.s. planes.

### Fractional atomic coordinates and isotropic or equivalent isotropic displacement parameters (Å^2^)

**Table:**

	*x*	*y*	*z*	*U*_iso_*/*U*_eq_	
O1	0.65312 (11)	0.12681 (7)	−0.07149 (10)	0.0503 (3)	
O2	0.63839 (11)	0.02849 (7)	0.10317 (10)	0.0584 (4)	
N1	0.84295 (18)	0.37076 (11)	0.11942 (17)	0.0817 (6)	
C1	0.74999 (16)	0.18513 (10)	−0.03978 (14)	0.0430 (4)	
C2	0.70930 (15)	0.24665 (10)	0.02407 (13)	0.0423 (4)	
C3	0.56268 (15)	0.23450 (9)	0.03502 (13)	0.0416 (4)	
H3	0.5515	0.2365	0.1156	0.050*	
C4	0.54406 (15)	0.14312 (9)	−0.00964 (14)	0.0432 (4)	
H4	0.4586	0.1385	−0.0614	0.052*	
C5	0.54745 (15)	0.07881 (10)	0.08555 (14)	0.0425 (4)	
C6	0.43803 (15)	0.08113 (10)	0.15584 (14)	0.0436 (4)	
C7	0.32474 (17)	0.13099 (11)	0.12818 (16)	0.0529 (5)	
H7	0.3158	0.1651	0.0635	0.063*	
C8	0.22520 (18)	0.13050 (12)	0.19565 (18)	0.0639 (5)	
H8	0.1487	0.1635	0.1759	0.077*	
C9	0.23861 (19)	0.08169 (13)	0.29133 (18)	0.0673 (6)	
H9	0.1720	0.0821	0.3376	0.081*	
C10	0.3498 (2)	0.03227 (13)	0.31933 (18)	0.0690 (6)	
H10	0.3584	−0.0011	0.3846	0.083*	
C11	0.44916 (18)	0.03135 (11)	0.25209 (16)	0.0557 (5)	
H11	0.5242	−0.0030	0.2716	0.067*	
C12	0.87832 (16)	0.16935 (11)	−0.07951 (14)	0.0466 (4)	
C13	0.9140 (2)	0.08775 (12)	−0.10240 (16)	0.0633 (5)	
H13	0.8558	0.0433	−0.0941	0.076*	
C14	1.0350 (2)	0.07193 (15)	−0.13726 (19)	0.0773 (6)	
H14	1.0591	0.0165	−0.1513	0.093*	
C15	1.12049 (19)	0.13654 (15)	−0.15166 (17)	0.0702 (6)	
H15	1.2021	0.1253	−0.1760	0.084*	
C16	1.08594 (19)	0.21745 (15)	−0.13026 (17)	0.0691 (6)	
H16	1.1441	0.2616	−0.1402	0.083*	
C17	0.96536 (17)	0.23435 (12)	−0.09402 (16)	0.0594 (5)	
H17	0.9425	0.2898	−0.0793	0.071*	
C18	0.47459 (14)	0.29991 (9)	−0.03105 (13)	0.0414 (4)	
C19	0.44746 (16)	0.29744 (10)	−0.14855 (15)	0.0488 (4)	
H19	0.4797	0.2527	−0.1876	0.059*	
C20	0.37323 (16)	0.36044 (11)	−0.20860 (16)	0.0565 (5)	
H20	0.3551	0.3577	−0.2878	0.068*	
C21	0.32609 (18)	0.42665 (12)	−0.15322 (19)	0.0635 (5)	
H21	0.2763	0.4692	−0.1942	0.076*	
C22	0.3523 (2)	0.43001 (12)	−0.03784 (19)	0.0745 (6)	
H22	0.3207	0.4754	0.0003	0.089*	
C23	0.42525 (19)	0.36704 (11)	0.02362 (17)	0.0646 (5)	
H23	0.4413	0.3699	0.1029	0.078*	
C24	0.78533 (16)	0.31484 (11)	0.07499 (16)	0.0513 (5)	

### Atomic displacement parameters (Å^2^)

**Table:**

	*U*^11^	*U*^22^	*U*^33^	*U*^12^	*U*^13^	*U*^23^
O1	0.0546 (7)	0.0452 (7)	0.0539 (7)	−0.0099 (6)	0.0172 (6)	−0.0127 (5)
O2	0.0528 (7)	0.0550 (8)	0.0674 (9)	0.0109 (6)	0.0097 (6)	0.0034 (6)
N1	0.0806 (12)	0.0659 (11)	0.0966 (14)	−0.0206 (10)	0.0077 (10)	−0.0269 (10)
C1	0.0468 (9)	0.0375 (9)	0.0443 (10)	−0.0054 (8)	0.0060 (8)	0.0005 (8)
C2	0.0430 (9)	0.0367 (9)	0.0463 (10)	−0.0020 (7)	0.0035 (7)	−0.0019 (8)
C3	0.0487 (9)	0.0389 (9)	0.0379 (9)	−0.0027 (7)	0.0094 (7)	−0.0017 (7)
C4	0.0427 (9)	0.0431 (9)	0.0445 (10)	−0.0044 (7)	0.0089 (8)	−0.0045 (8)
C5	0.0413 (9)	0.0364 (9)	0.0482 (10)	−0.0045 (8)	0.0021 (8)	−0.0038 (8)
C6	0.0445 (9)	0.0393 (9)	0.0466 (10)	−0.0038 (8)	0.0059 (8)	0.0007 (8)
C7	0.0467 (10)	0.0562 (11)	0.0559 (11)	0.0022 (9)	0.0081 (9)	0.0100 (9)
C8	0.0515 (11)	0.0690 (13)	0.0716 (14)	0.0094 (10)	0.0111 (10)	0.0070 (11)
C9	0.0635 (13)	0.0747 (13)	0.0692 (14)	−0.0002 (11)	0.0281 (11)	0.0056 (11)
C10	0.0703 (13)	0.0761 (14)	0.0635 (13)	0.0015 (11)	0.0192 (11)	0.0218 (11)
C11	0.0564 (11)	0.0520 (11)	0.0589 (12)	0.0038 (9)	0.0092 (9)	0.0108 (9)
C12	0.0482 (10)	0.0476 (10)	0.0445 (10)	0.0023 (8)	0.0084 (8)	0.0031 (8)
C13	0.0719 (13)	0.0543 (12)	0.0687 (14)	0.0009 (10)	0.0264 (10)	−0.0070 (10)
C14	0.0773 (14)	0.0734 (15)	0.0873 (17)	0.0169 (13)	0.0319 (12)	−0.0070 (12)
C15	0.0540 (12)	0.1010 (18)	0.0580 (13)	0.0167 (13)	0.0167 (10)	0.0003 (12)
C16	0.0489 (11)	0.0859 (16)	0.0743 (15)	−0.0054 (11)	0.0151 (10)	0.0106 (12)
C17	0.0522 (11)	0.0547 (11)	0.0727 (14)	0.0007 (9)	0.0144 (10)	0.0062 (10)
C18	0.0399 (9)	0.0406 (9)	0.0450 (10)	−0.0033 (7)	0.0106 (7)	−0.0037 (8)
C19	0.0503 (10)	0.0482 (10)	0.0487 (11)	0.0018 (8)	0.0100 (8)	−0.0028 (8)
C20	0.0543 (11)	0.0626 (12)	0.0510 (11)	−0.0020 (10)	0.0032 (9)	0.0056 (10)
C21	0.0557 (11)	0.0582 (12)	0.0752 (15)	0.0094 (10)	0.0050 (10)	0.0093 (11)
C22	0.0857 (15)	0.0630 (13)	0.0764 (16)	0.0308 (12)	0.0173 (12)	−0.0053 (11)
C23	0.0802 (13)	0.0641 (12)	0.0503 (12)	0.0166 (11)	0.0125 (10)	−0.0083 (10)
C24	0.0503 (10)	0.0459 (10)	0.0564 (11)	−0.0014 (9)	0.0047 (9)	−0.0047 (9)

### Geometric parameters (Å, º)

**Table:**

O1—C1	1.3523 (18)	C11—H11	0.9300
O1—C4	1.4352 (18)	C12—C13	1.377 (2)
O2—C5	1.2064 (17)	C12—C17	1.378 (2)
N1—C24	1.140 (2)	C13—C14	1.368 (3)
C1—C2	1.335 (2)	C13—H13	0.9300
C1—C12	1.464 (2)	C14—C15	1.363 (3)
C2—C24	1.403 (2)	C14—H14	0.9300
C2—C3	1.514 (2)	C15—C16	1.359 (3)
C3—C18	1.502 (2)	C15—H15	0.9300
C3—C4	1.539 (2)	C16—C17	1.375 (2)
C3—H3	0.9800	C16—H16	0.9300
C4—C5	1.516 (2)	C17—H17	0.9300
C4—H4	0.9800	C18—C23	1.375 (2)
C5—C6	1.481 (2)	C18—C19	1.379 (2)
C6—C11	1.377 (2)	C19—C20	1.376 (2)
C6—C7	1.384 (2)	C19—H19	0.9300
C7—C8	1.376 (2)	C20—C21	1.359 (3)
C7—H7	0.9300	C20—H20	0.9300
C8—C9	1.362 (3)	C21—C22	1.354 (3)
C8—H8	0.9300	C21—H21	0.9300
C9—C10	1.363 (3)	C22—C23	1.377 (3)
C9—H9	0.9300	C22—H22	0.9300
C10—C11	1.373 (2)	C23—H23	0.9300
C10—H10	0.9300		
			
C1—O1—C4	108.15 (11)	C6—C11—H11	119.9
C2—C1—O1	112.48 (14)	C13—C12—C17	118.86 (16)
C2—C1—C12	132.09 (15)	C13—C12—C1	119.57 (16)
O1—C1—C12	115.42 (13)	C17—C12—C1	121.57 (16)
C1—C2—C24	127.47 (15)	C14—C13—C12	120.16 (19)
C1—C2—C3	110.19 (13)	C14—C13—H13	119.9
C24—C2—C3	122.34 (14)	C12—C13—H13	119.9
C18—C3—C2	111.91 (12)	C15—C14—C13	120.7 (2)
C18—C3—C4	115.79 (13)	C15—C14—H14	119.7
C2—C3—C4	99.09 (12)	C13—C14—H14	119.7
C18—C3—H3	109.9	C16—C15—C14	119.74 (18)
C2—C3—H3	109.9	C16—C15—H15	120.1
C4—C3—H3	109.9	C14—C15—H15	120.1
O1—C4—C5	109.25 (12)	C15—C16—C17	120.32 (19)
O1—C4—C3	106.39 (12)	C15—C16—H16	119.8
C5—C4—C3	112.63 (13)	C17—C16—H16	119.8
O1—C4—H4	109.5	C16—C17—C12	120.24 (18)
C5—C4—H4	109.5	C16—C17—H17	119.9
C3—C4—H4	109.5	C12—C17—H17	119.9
O2—C5—C6	121.97 (15)	C23—C18—C19	117.96 (15)
O2—C5—C4	120.00 (14)	C23—C18—C3	120.79 (15)
C6—C5—C4	118.02 (14)	C19—C18—C3	121.13 (14)
C11—C6—C7	118.72 (16)	C20—C19—C18	120.66 (16)
C11—C6—C5	118.71 (15)	C20—C19—H19	119.7
C7—C6—C5	122.57 (15)	C18—C19—H19	119.7
C8—C7—C6	120.46 (17)	C21—C20—C19	120.59 (18)
C8—C7—H7	119.8	C21—C20—H20	119.7
C6—C7—H7	119.8	C19—C20—H20	119.7
C9—C8—C7	120.05 (18)	C22—C21—C20	119.37 (18)
C9—C8—H8	120.0	C22—C21—H21	120.3
C7—C8—H8	120.0	C20—C21—H21	120.3
C8—C9—C10	119.96 (18)	C21—C22—C23	120.77 (18)
C8—C9—H9	120.0	C21—C22—H22	119.6
C10—C9—H9	120.0	C23—C22—H22	119.6
C9—C10—C11	120.62 (18)	C18—C23—C22	120.63 (18)
C9—C10—H10	119.7	C18—C23—H23	119.7
C11—C10—H10	119.7	C22—C23—H23	119.7
C10—C11—C6	120.17 (17)	N1—C24—C2	177.0 (2)
C10—C11—H11	119.9		
			
C4—O1—C1—C2	−9.45 (18)	C8—C9—C10—C11	0.2 (3)
C4—O1—C1—C12	169.86 (13)	C9—C10—C11—C6	0.6 (3)
O1—C1—C2—C24	177.16 (15)	C7—C6—C11—C10	−0.6 (3)
C12—C1—C2—C24	−2.0 (3)	C5—C6—C11—C10	179.74 (17)
O1—C1—C2—C3	−3.50 (19)	C2—C1—C12—C13	153.06 (19)
C12—C1—C2—C3	177.34 (16)	O1—C1—C12—C13	−26.1 (2)
C1—C2—C3—C18	−109.07 (15)	C2—C1—C12—C17	−26.5 (3)
C24—C2—C3—C18	70.31 (19)	O1—C1—C12—C17	154.34 (15)
C1—C2—C3—C4	13.56 (16)	C17—C12—C13—C14	1.0 (3)
C24—C2—C3—C4	−167.06 (15)	C1—C12—C13—C14	−178.61 (17)
C1—O1—C4—C5	−103.83 (13)	C12—C13—C14—C15	−1.1 (3)
C1—O1—C4—C3	18.00 (16)	C13—C14—C15—C16	0.6 (3)
C18—C3—C4—O1	101.41 (15)	C14—C15—C16—C17	0.1 (3)
C2—C3—C4—O1	−18.39 (15)	C15—C16—C17—C12	−0.2 (3)
C18—C3—C4—C5	−138.92 (14)	C13—C12—C17—C16	−0.3 (3)
C2—C3—C4—C5	101.28 (14)	C1—C12—C17—C16	179.27 (16)
O1—C4—C5—O2	4.62 (19)	C2—C3—C18—C23	−103.03 (18)
C3—C4—C5—O2	−113.38 (16)	C4—C3—C18—C23	144.42 (16)
O1—C4—C5—C6	−176.36 (12)	C2—C3—C18—C19	72.97 (17)
C3—C4—C5—C6	65.64 (17)	C4—C3—C18—C19	−39.6 (2)
O2—C5—C6—C11	7.9 (2)	C23—C18—C19—C20	0.0 (2)
C4—C5—C6—C11	−171.09 (15)	C3—C18—C19—C20	−176.12 (14)
O2—C5—C6—C7	−171.69 (15)	C18—C19—C20—C21	0.5 (3)
C4—C5—C6—C7	9.3 (2)	C19—C20—C21—C22	−0.3 (3)
C11—C6—C7—C8	−0.1 (3)	C20—C21—C22—C23	−0.4 (3)
C5—C6—C7—C8	179.46 (16)	C19—C18—C23—C22	−0.7 (3)
C6—C7—C8—C9	1.0 (3)	C3—C18—C23—C22	175.45 (16)
C7—C8—C9—C10	−1.0 (3)	C21—C22—C23—C18	0.9 (3)

### Hydrogen-bond geometry (Å, º)

**Table:**

*D*—H···*A*	*D*—H	H···*A*	*D*···*A*	*D*—H···*A*
C11—H11···N1^i^	0.93	2.63	3.487 (2)	154
C21—H21···O2^ii^	0.93	2.58	3.277 (2)	132

Symmetry codes: (i) −*x*+3/2, *y*−1/2, −*z*+1/2; (ii) *x*−1/2, −*y*+1/2, *z*−1/2.
