# Supplementary figures and images for: Crystal structure of 5-benzoyl-2,4-di­phenyl-4,5-di­hydro­furan-3-carbo­nitrile
Source: Acta Crystallogr E Crystallogr Commun. 2015 Aug 15;71(Pt 9):o663–4. doi: 10.1107/S2056989015014887 (PMC4555400; doi:10.1107/S2056989015014887)

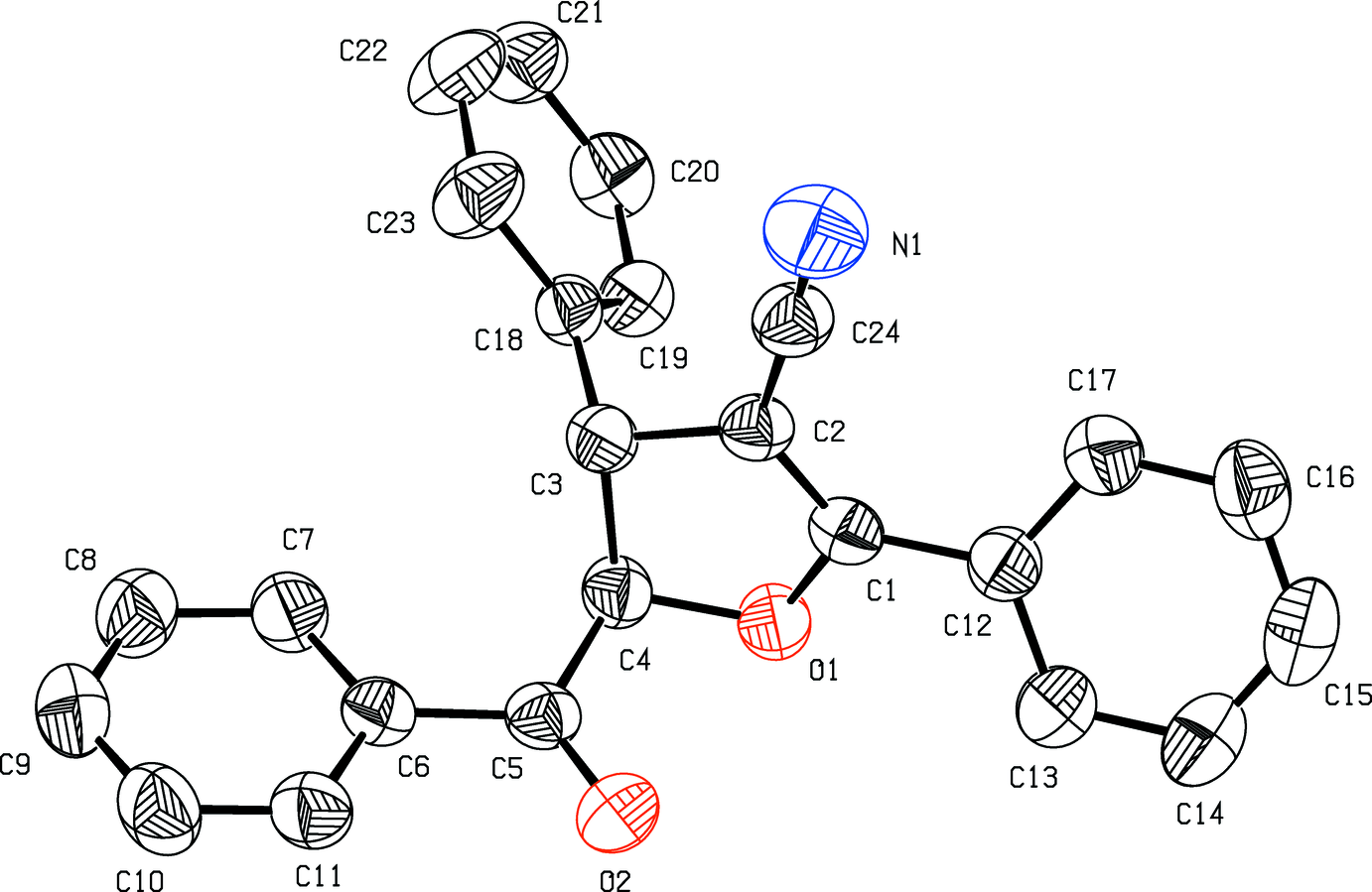

Supplement: Supplementary file 4 [file e-71-0o663-fig1.tif]

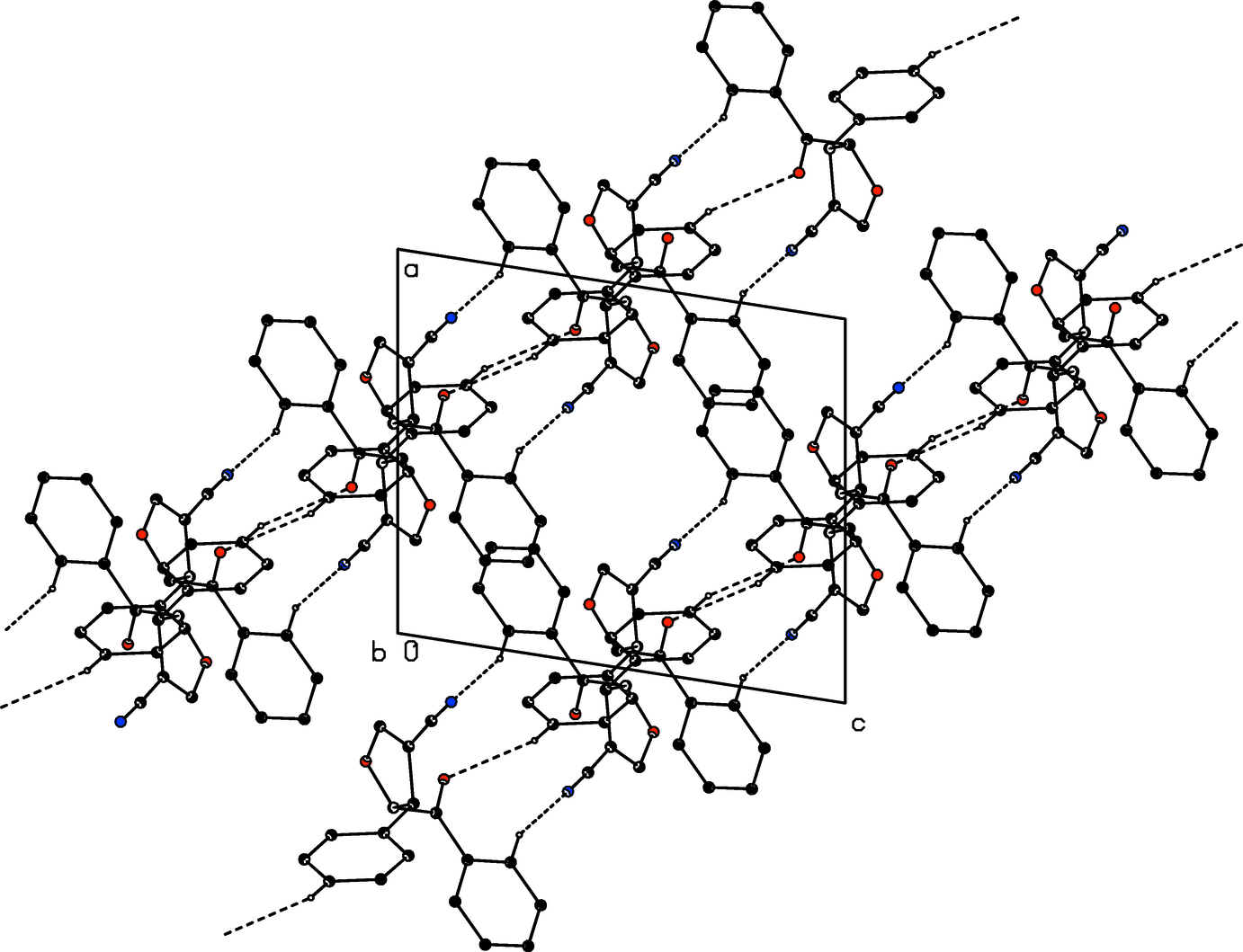

Supplement: Supplementary file 5 [file e-71-0o663-fig2.tif]

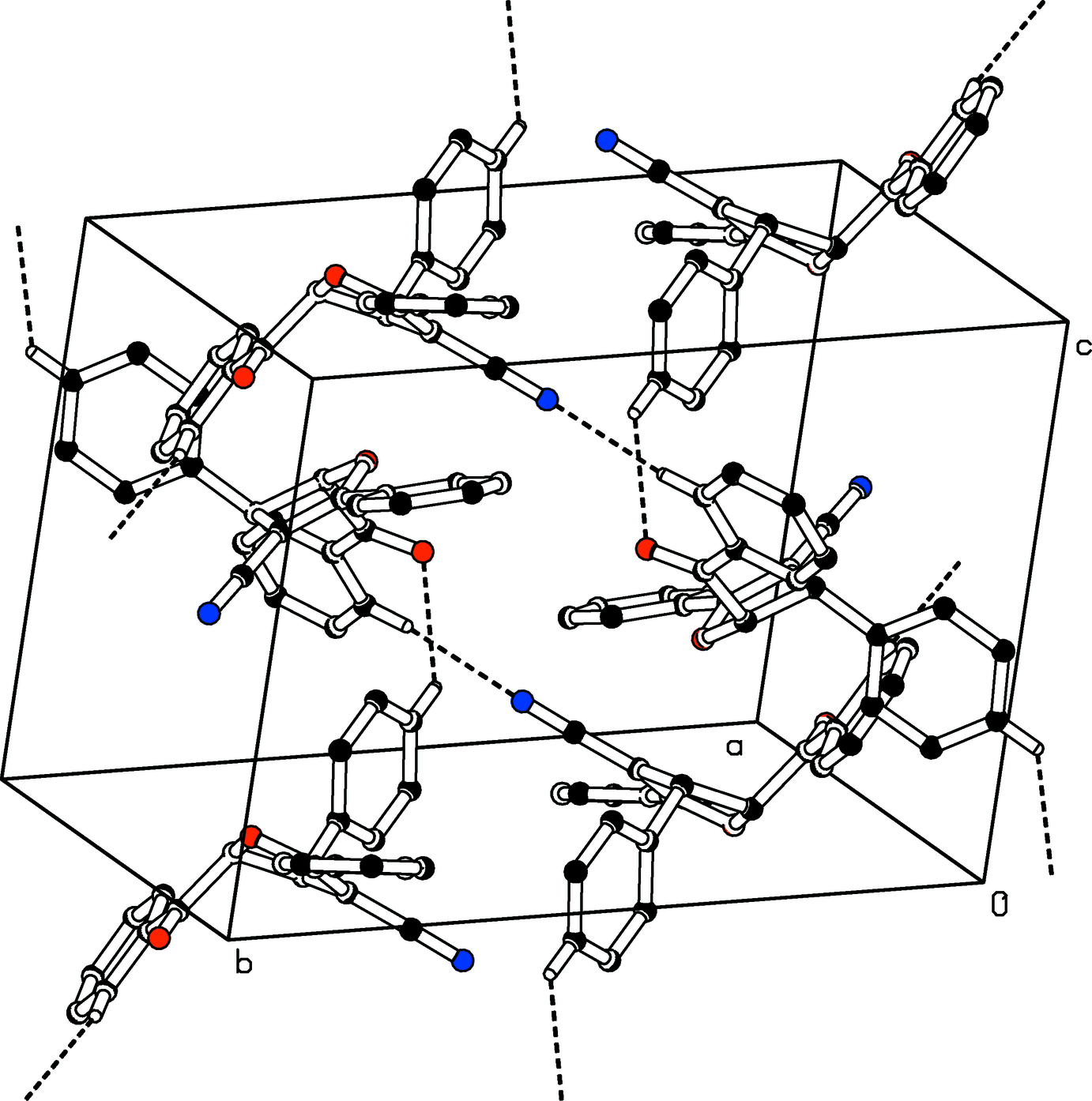

Supplement: Supplementary file 6 [file e-71-0o663-fig3.tif]
